# The role of silica-containing agro-industrial waste as reinforcement on physicochemical and thermal properties of polymer composites

**DOI:** 10.1016/j.heliyon.2020.e03550

**Published:** 2020-03-11

**Authors:** Samsul Rizal, H.M. Fizree, Md. Sohrab Hossain, Ikramullah, Deepu A. Gopakumar, Eunice Chong Wan Ni, H.P.S. Abdul Khalil

**Affiliations:** a Department of Mechanical Engineering, Universitas Syiah Kuala, Banda Aceh 23111, Indonesia; b School of Industrial Technology, Universiti Sains Malaysia, 11800 Penang, Malaysia

**Keywords:** Materials science, Agro-industrial waste, Inorganic SiO2, Oil palm boiler ash, Micro filler, Epoxy composites

## Abstract

This study was conducted to determine the influence of the oil palm boiler ash (OPBA) reinforcement on the microstructural, physical, mechanical and thermal properties of epoxy polymer composites. The chemical composition analysis of OPBA revealed that it contains about 55 wt.% of SiO_2_ along with other metallic oxides and elements. The surface morphology of OPBA showed angular and irregular shapes with porous structures. The influence of OPBA as a reinforcement in epoxy composite was studied with varying filler loadings (10–50 wt.%) and different particle sizes (50–150 μm). The result showed that the incorporation of OPBA in composites has improved the physical, mechanical and thermal properties of the epoxy matrix. The highest physical and mechanical properties of fabricated composites were attained with 30 wt.% loading and size of 50 μm. Also, thermal stability and the percentage of char residue of the composite increased with increasing filler loading. Furthermore, the contact angle of OPBA reinforced epoxy composites increased with the increase of filler loading. The lowest value of the contact angle was obtained at 30 wt.% of filler loading with the OPBA particle size of 50 μm. The finding of this study reveals that the OPBA has the potential to be used as reinforcement or filler as well as an alternative of silica-based inorganic fillers used in the enhancement of mechanical, physical and thermal properties of the epoxy polymer composite.

## 1 Introduction

There is an increasing concern on the sustainable utilization of agricultural waste to minimize the environmental burden as well as preserve the ecosystem. The oil palm is one of the most successful agro-industrial crops and the plantations are advancing rapidly worldwide. The use of oil palm has a wide range of applications because every part of the plant has its unique properties [1]. A huge amount of solid waste such as oil palm fibre, palm kernel shell and oil palm empty fruit bunch have been produced by the palm oil industries worldwide [2]. The palm kernel shell and fibre can be utilized as fuel for the oil palm mill boiler used in generating steam and electricity [3].

Palm oil is the most consumed edible vegetable oil. The palm oil industry is the active contributor to the gross domestic product (GDP) of Indonesia and Malaysia [1]. However, the palm oil industry is facing massive criticism in recent years because of its effect on the environment [4]. One of the main criticization received by the palm oil industry is the improper disposal practices of the solid waste generated during processing the oil palm fresh fruits bunch for palm oil production. It is being estimated that about 51.19 million tonnes (Mt) of oil palm waste including 4 Mt of oil palm boiler ash is generated by the Malaysian palm oil industry in 2018 [4]. Amongst the generated waste, the oil palm boiler ash (OPBA) is at the forefront of criticism due to its carcinogenic characteristics and bio-accumulative tendency [5]. The safe disposal cost of this OPBA is costly, hitting as high as $5 to $50 from developing to developed countries [5]. Thus, it urges an innovative approach to transform this ash into a valuable end product to minimize palm oil production cost as well as reducing the negative impact on the environment.

Epoxy polymeric composite has wide ranges of industrial applications from packaging to electronics industries owing to its distinct desirable properties including superior thermal stability, low shrinkage, excellent chemical resistance and high stiffness [6]. However, the poor mechanical properties and fragility of the epoxy matrix have restricted its performances. Studies have been utilized various inorganic elements/compounds as reinforcements to enhance the physical, mechanical and thermal properties of the epoxy matrix [6,7,8]. However, it is being reported that silica is one of the most influential and ideal reinforcement materials among the various inorganic fillers [8]. Moreover, silica is the most utilized inorganic materials as reinforcement in the polymeric composites because of its proven ability to improve the mechanical properties of a polymeric composite including fracture toughness, impacts strength and flexural properties [8].

Chemical properties analyses of OPBA reveals that the oil palm boiler ash contained a significant amount of silica (about 53 wt.%) along with other valuable inorganic particles [9]. Thus, it is of considerable interest to utilize OPBA as reinforcement in epoxy polymeric composite to enhance mechanical properties. The utilization of these waste materials in the composite matrix would benefit the palm oil industry in many ways, including minimizing waste load dispose to landfills, reduce solid waste disposal cost and enhance sustainability. Hence, in this study, OPBA was utilized as a reinforcement in the polymer matrix. The OPBA was collected from palm oil mill and ground to particle size 50–150 μm. The physicochemical properties of microstructured OPBA were determined using X-Ray Fluorescence (XRF), Fourier Transform Infrared Spectroscopy (FT-IR), Brunauer–Emmett–Teller (BET) and scanning Electron Microscope (SEM). Subsequently, the OPBA was used as reinforcement in epoxy composite with varying percentage loading (10–50%) and particle sizes. Finally, the effect of OPBA as reinforcement in the epoxy matrix was analysed for an increase in the physical, chemical, morphological and thermal properties of the composites.

## 2 Materials and methods

### 2.1 Materials

Epoxy resin D.E.R 331, used as the composite material, was supplied by the Zarm Scientific and Supplies Sdn. Bhd, Malaysia. Isophorone diamine used as an epoxy hardener was obtained from ZARAM scientific, Malaysia. Benzyl alcohol purchased from Sigma-Aldrich, Malaysia was used to reduce viscosity and to facilitate the good dispersion process of OPBA in the epoxy-polyamide matrix.

### 2.2 Preparation of OPBA

**Figure 1:**
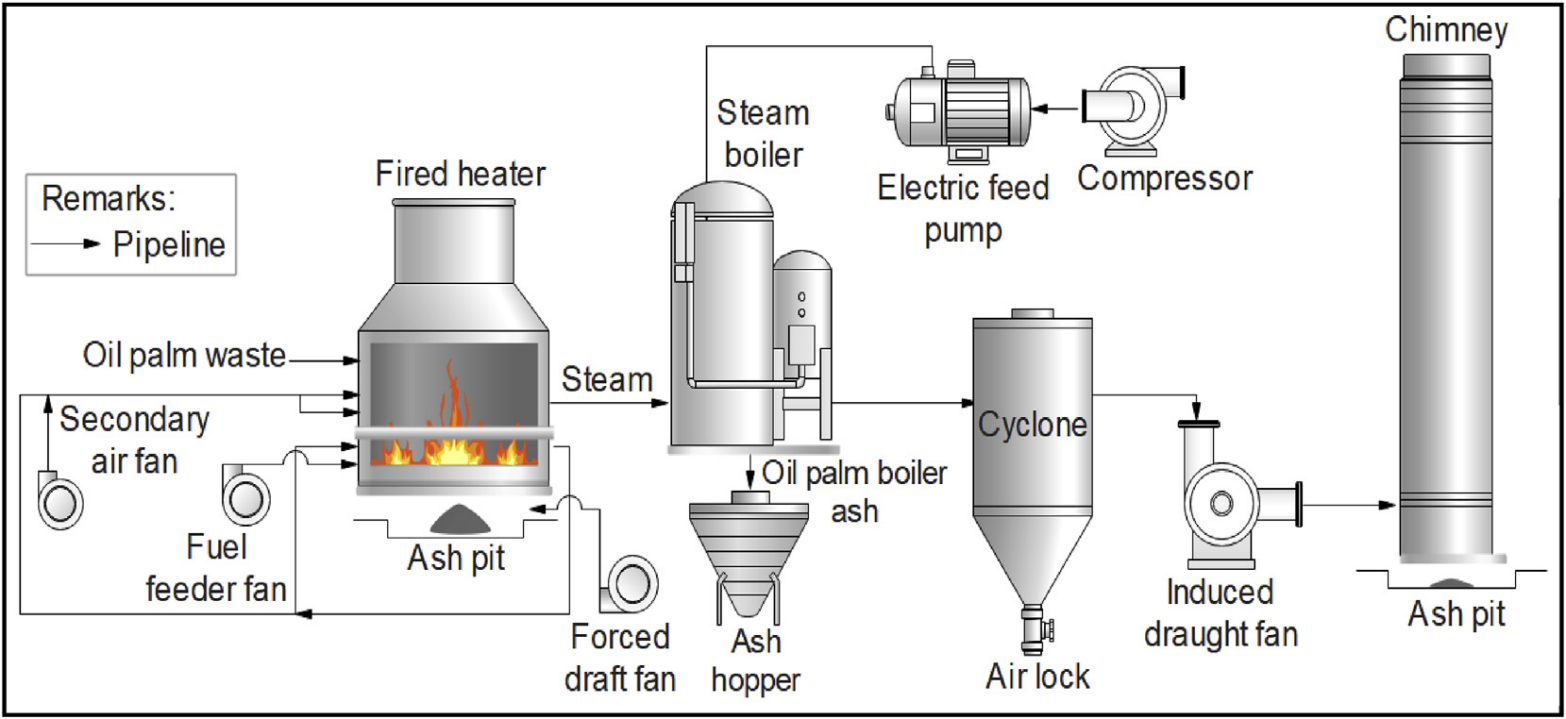
Schematic diagram of the production of oil palm boiler ash (OPBA).

The oil palm boiler ash (OPBA) was collected from United Palm Oil Mill, Nibong Tebal Penang, Malaysia. The OPBA was collected from the ash hopper of the oil palm steam boiler, as shown in Figure 1. The OPBA collected was dried at 105 °C for 24 h to eliminate moisture. The dried OPBA was then sieved using 60 mesh size sieves to separate impurities. Then, it was grounded by using a microfine grinder, (MF 10 -IKA®, Germany) at 6500 rpm. Subsequently, the grounded OPBA was sieved to the particle size of 50 μm, 100 μm, and 150 μm. The sieved OPBA was conditioned in an oven at 250 °C for 24 h to prevent agglomeration and stored in a dry place.

### 2.3 Characterization of OPBA

The chemical compositions of raw OPBA were determined using X-Ray Fluorescence (XRF) (PANalytical model Axios MAX -4kW). The particle size of OPBA was examined using dynamic light scattering with 532 nm laser (Malvern Zetasizer Nano Series). The surface morphology of the OPAB was inspected using a field emission scanning electron microscope (FE-SEM-Leo Supra, 50 VP, Carl Ziess, SMT, Germany). Approximately, 1 mg of OPBA was coated with gold powder and mounted onto sample holder using double-sided adhesive tape. The FE-SEM micrographs of raw OPBA and microstructure OPBA were taken with an acceleration voltage of 5 kV.

Fourier Transform Infrared Spectroscopy (FT-IR) spectrophotometer (Perkin-Elmer, PC1600, USA) analysis of OPBA was performed using a spectra range of 300 and 4000 cm^−1^. The density of OPBA was determined by using the Digital Density meter (Gas pycnometer-AccuPyc 1330), conducted at temperature 22.8 °C and pressure of 134 kPa. The specific surface area of the OPBA was determined by the Brunauer–Emmett–Teller (BET) equipped with Quantachrome Nova Win2© 1994–2002 instrument.

### 2.4 Fabrication of OPBA filled epoxy polymer composite

The influence of OPBA loading on the properties of epoxy composite fabrication was determined with varying OPBA particle sizes (50 μm, 100 μm, and 150 μm) and filler loadings (10–50 wt.%). 10% of diluents (benzyl alcohol) and OPBA were mixed under the vigorous mechanical stirrer at 3000 rpm for 30 min. Later, 50 parts curing agent (polyamine hardener) in 100 parts epoxy by weight basis was measured into the mixture and stirred for 10 min at 170 rpm. Subsequently, the mixture was placed in a vacuum chamber for 15 min to remove bubbles and cast on a stainless-steel mould. The moulded composite samples were then cured at 105 °C for 1 h in a hot press (Gotech Hot Press-Gt-7014; Taiwan) at 1.379 × 10^3^ kPa. Post curing of the fabricated composite was dried in an oven at 105 °C for 30 min.

### 2.5 Characterizations of OPBA filled epoxy polymer composite

#### 2.5.1 Physical properties

The density of microstructure OPBA filled epoxy composites was determined, following the standard method for apparent density provided by the American Society for Testing and Materials (ASTM-D1895) [10]. The density of the composite was calculated using Eq. (1). The experiments were conducted in triplicate and the average value of triplicate experimental runs were reported.

(1) $$\text{Density}\left({\text{gcm}}^{-3}\right)=\frac{\text{m}}{\text{v}}$$

where *m* is the mass of the composites (g) and *v* is the volume of composites (cm^3^). The mass and the volume of the composite were determined using an analytical balance (Mettler 5000) and digital veneer calliper (Mitutoyo), respectively. The composites were dried in an oven at 50 °C for 24 h and cooled in a desiccator contained granulated silica gel before determining the mass and volume of the composite. The void content of the composite was calculated by the following equation [11].

(2) $$\text{Void Content }\left(\text{\%}\right)=\frac{{\text{ρ}}_{\text{theoretical}}-{\text{ρ}}_{\text{practical}}}{{\text{ρ}}_{\text{theoretical}}}\times 100$$

where *p*_*theortical*_ is the theoretical density and _*practical*_ is the actual density of the epoxy polymer composite. The *p*_*theortical*_ can be calculated using Eq. (3).

(3) $$\rho_{\text{theoretical}}=\frac{1}{\frac{w_{f}}{\rho_{f}}+\frac{w_{m}}{\rho_{m}}}$$

where W_f_ is the weight fraction of OPBA filler, W_m_ is the weight fraction of the matrix, *p*_*f*_ refers to the filler density and *p*_m_ refers to the matrix density.

#### 2.5.2 Mechanical properties

The mechanical properties such as tensile test, flexural test and impact strength of OPBA filled epoxy composite was determined based on ASTM standards. The tensile test of microstructure OPBA filled epoxy composite was conducted at ambient temperature and relative humidity of 50% according to ASTM-D3039 [12]. The testing speed was fixed at 5 mm/min and the gauge length was set at 60 mm. The dog bone shape of the tested composite was in 120 mm × 15 mm × 3 mm ± 3 mm^3^ dimension. The flexural test was determined according to ASTM-D790 [13] at a speed of 2 mm/min using a Universal Instron 5582 machine. The sample shape was rectangular with a dimension 160 mm × 20 mm×3mm ±3mm. Izod notched impact test was performed according to the ASTM-D256 method using the Geotech testing machine [14]. The tested composite dimension was 120 mm × 15 mm × 3 mm ± 3 mm^3^. Five (5) samples each were tested for tensile, flexural and impacts test and the results were averaged.

#### 2.5.3 Thermogravimetric analysis (TGA)

The thermogravimetric analysis of neat epoxy and OPBA filled epoxy composite was conducted using a Perkin Elmer thermal gravimetric analyzer (TGA-6). Neat epoxy and OPBA filled epoxy composite of 4–5 mg were tested to determine the mass lost concerning temperature from 30 to 800 °C at a heating rate of 20 °C/min under nitrogen atmosphere.

#### 2.5.4 Contact angle measurement

The static contact angle of the OPBA filled epoxy composites was determined using the sessile drop technique CAM 101 KSV Instrument. A drop of test liquid was uniformly dropped on the surface of the epoxy composite, and the image was taken for every 5 s at a speed of 5 frames per second and analysed using CAM 101 KSV Instrument. The test was repeated for 5 times and the average result was taken.

## 3 Results and discussion

### 3.1 Characterization of OPBA

The chemical composition of the OPBA was determined by X-Ray Fluorescence (XRF), as presented in Table 1. The analysis showed that the major chemical elements present in the OPBA were SiO_2_ (54.91%), K_2_O (10.67%), CaO (10.13%), Fe_2_O_3_ (5.69%), SO_3_ (4.92%), P_2_O_5_ (4.17%) Al_2_O_3_ (3.31&) and MgO (3.49%). Among the various inorganic compounds detected in OPBA were SiO_2_, CaO, TiO_2,_ and Al_2_O_3_. These compounds have been frequently used as fillers in composite owing to their outstanding mechanical, thermal and physical properties [8,9,15]. Therefore, the presence of these inorganic compounds in OPBA suggested that the OPBA could be utilized as a filler or reinforcement in polymer composite. Also, a trace of some metallic elements found in OPBA as reported in Table 1 corroborates its use as filler to enhance the mechanical and thermal properties of polymer composites.

**Table 1:** Inorganic chemical composition and trace elements in OPBA.

Major components	Percentage (%)	Trace elements	μg/g
SiO_2_	54.91	Ba	ND∗
TiO_2_	0.2	Br	50
Al_2_O_3_	3.31	Cr	110
Fe_2_O_3_	5.69	Cu	400
MnO	0.12	Ni	ND
MgO	3.49	Rb	380
CaO	10.13	Sm	ND
Na_2_O	0.36	Sr	150
K_2_O	10.67	Y	10
P_2_O_5_	4.17	Zn	150
SO_3_	4.92	Zr	180
Cl	1.89	-	-

∗ (ND=Not Detected).

**Figure 2:**
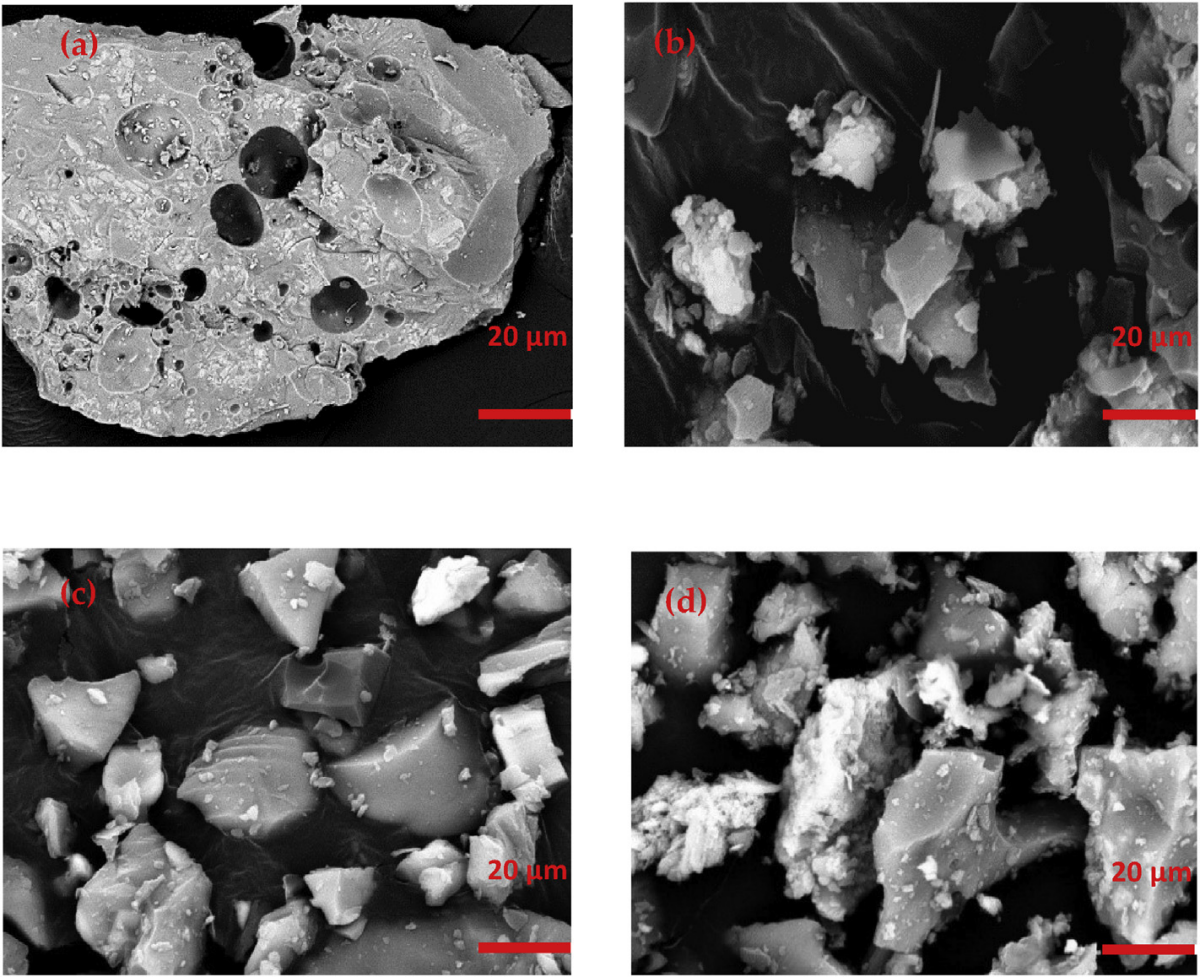
Morphologies of OPBA (5000 × magnification); (a) raw (b) 50 μm, (c) 100 μm and (d) 150 μm.

The morphological alteration of the raw OPBA was observed under FE-SEM and the images were presented in Figure 2. It was found that the raw OPBA showed porous-like angular and irregular shapes particles. The FE-SEM images of microstructure OPBA also showed that OPBA particles were in irregular shapes but crushed. Interestingly, the porosity of the OPBA particle was hardly found on the surface of microstructure OPBA images. This is because of the size of the microstructure OPBA which is smaller compared to the raw OPBA.

**Figure 3:**
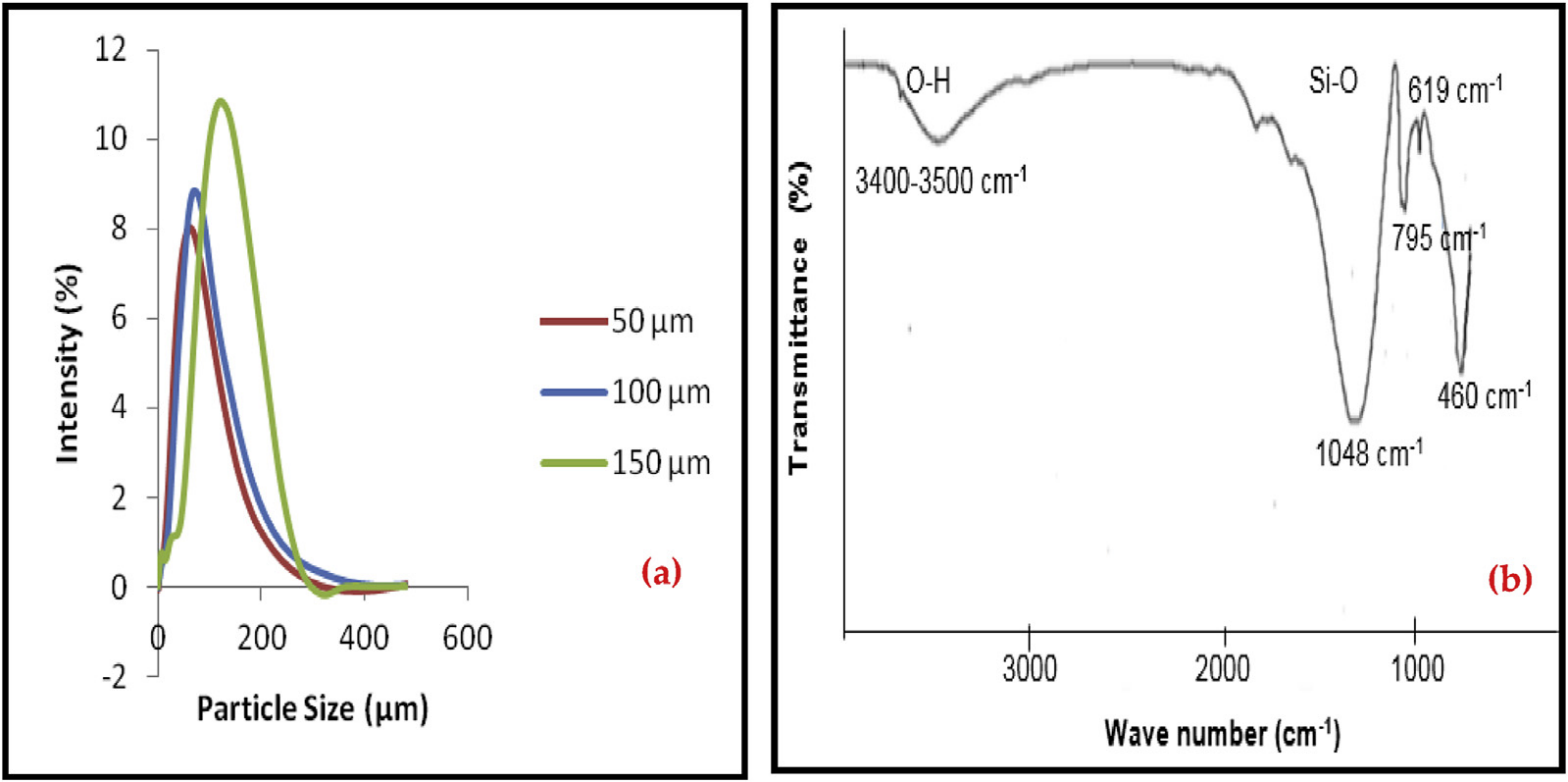
(a) Particle size distribution of OPBA, (b). FT-IR transmission spectrum of Raw OPBA.

Particle size is an important analysis for characterization and vital parameters in optimizing the performances of composites produced. A Particle Size Zetasizer Analyzer was used to determine the particle size distribution of the microstructure OPBA fillers as shown in Figure 3 and Table 2. The average diameter of OPBA is enhanced with increasing particle sizes while the surface area decreased with increasing particle sizes. This result indicated that the higher surface area is due to the increase of the surface-to-volume ratio [16]. However, there was a negligible impact observed on the OPBA density with decreasing particle sizes.

**Table 2:** Particle size, density, surface area and aspect ratio of OPBA at different particle sizes.

Particle Size (μm)	Average Diameter (μm)	Density (g/m^3^)	Surface Area (m^2^/g)	Aspect Ratio
50	50.46	2.559	4.5337	0.99
100	71.23	2.542	1.0594	1.30
150	157.26	2.522	0.0721	1.55

Figure 3 (b) displays the FT-IR transmission spectrum of the raw OPBA. The presence of SiO_2_ in raw OPBA has been proven via Fourier transform infrared (FT-IR) spectroscopy. A discernible band at 619 cm^−1^ is assigned to the crystalline cristobalite [17]. The characteristic vibrational bands noticed at 1048, 795, and 460 cm^−1^ correspond to the stretching, bending and out of plane deformation of Si–O bonds, respectively. The FT-IR spectra of OPBA observed at 1048 cm^−1^ implies the stoichiometric of SiO_2_ structure [18]. Besides, the presence of bonded and non-bonded –OH groups were detected in between the range of 3300–3500 cm^−1^, and this can be attributed to the presence of silanol hydroxyl groups on the surface of oil palm ash and the stretching vibration of –OH groups [17].

### 3.2 Physical properties of OPBA filled epoxy polymer composite

The influence of incorporating OPBA on the physical properties of the epoxy matrix was studied with varying particle sizes (50–150 μm) and filler loadings (10, 20, 30, 50 wt.%) as presented in Table 3. The result showed that the theoretical, actual densities and void content of the neat epoxy composites were 1.12 g/cm^3^, 1.096 g/cm^3,^ and 1.378%, respectively. The highest density of OPBA filled epoxy composite (1.338 g/cm^3^) was recorded at a particle size of 50 μm and 50 wt.% filler loading. The actual density of the epoxy composites increased with increasing incorporation of microstructured OPBA filler loading (wt.%). However, it decreases with the particle sizes from 50 to 150 μm. The increase of density with increasing filler loading is mainly attributed to the incorporation of high-density microstructured OPBA while the reduction in density with increasing particle size, can be attributed to good distribution of smaller size fillers. As smaller filler was incorporated into the matrix, it filled up the void of the matrix. The percentage of silica increased in per unit volume of the composite and this was reported by Jiang and Yuan [19].

**Table 3:** Physical properties of OPBA reinforced epoxy polymer composite.

Filler loading (wt.%)	Particle size								
	50 (μm)			100 (μm)			150 (μm)		
	Measured density (g/cm^3^)	Theoretical density (g/cm^3^)	Void Content (%)	Measured density (g/cm^3^)	Theoretical density (g/cm^3^)	Void Content (%)	Measured density (g/cm^3^)	Theoretical density (g/cm^3^)	Void Content (%)
10	1.157	1.172	1.279	1.1463	1.1716	2.134	1.137	1.171	2.903
20	1.208	1.227	1.548	1.1961	1.2267	2.446	1.187	1.226	3.181
30	1.255	1.279	1.876	1.2438	1.2775	2.662	1.234	1.276	3.291
40	1.297	1.326	2.187	1.2868	1.3246	2.87	1.278	1.323	3.401
50	1.338	1.37	2.335	1.3279	1.3683	2.997	1.318	1.366	3.513
Neat Epoxy	1.096	1.112	1.378						

**Figure 4:**
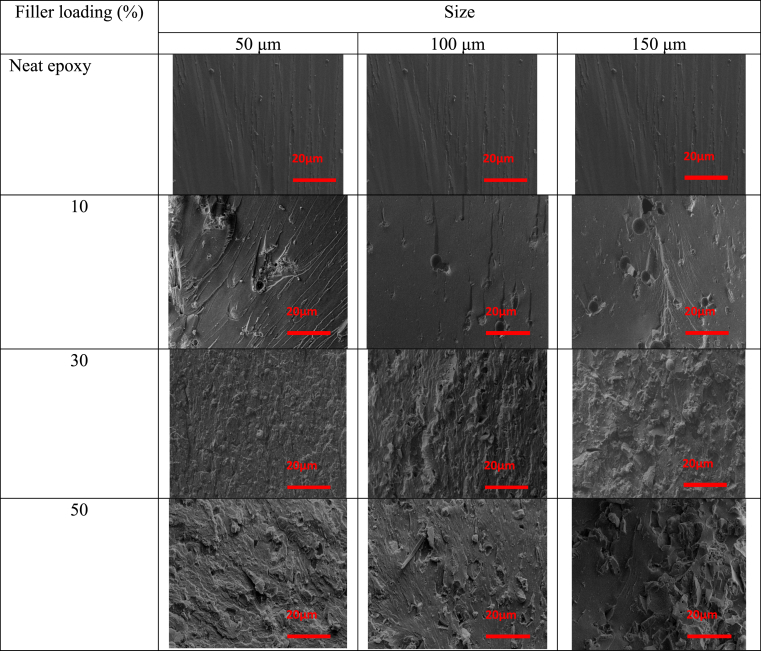
Fractured surfaces of epoxy polymer composite incorporated with OPBA with varying loadings and sizes (100 × magnification).

The actual density of OPBA filled epoxy composites was different from the theoretical density of the composites, calculated using Eq. (1) (Table 3). The difference between calculated and actual density values of OPBA filled epoxy composites could be due to the presence of voids in the composites. It was found that the void content of epoxy composite increased with increasing filler loading and particle size. The highest void content of OPBA filled epoxy composites was 3.513%, which was achieved with 50 wt.% filler loading at 150 μm particle size. Generally, void formation in polymer composites can be attributed to entrapped air bubbles within the epoxy matrix and volatile residual solvent from the curing of the resin [20]. The void formation may influence the impact of curing pressure, resin, and temperature during the fabrication of composites [21]. The presence of voids can influence the mechanical properties of the epoxy composites. Samples with lower void contents of the composites often show better susceptibility to weathering resistance and water penetration. In this case, smaller size filler at lower filler loading showed lower void content (%). This could be due to lesser filler pull-out from the matrix compared to those with bigger size and higher filler loading. This is further confirmed by FESEM shown in Figure 4. However, the higher void content observed in the OPBA reinforced epoxy composite (except for 50μm, 10 wt.% OPBA loading) compared to the neat epoxy could be due to agglomeration and uneven distribution of fillers, which is in agreement with the result from FE-SEM.

### 3.3 Mechanical properties of OPBA filled epoxy polymer composite

The tensile and flexural properties of OPBA reinforcement in epoxy polymer composite was determined with varying filler loadings (10–50 wt.%) and particle sizes (50–150 μm), as presented in Table 4. It was found that tensile strength (MPa), tensile modulus (GPa) and percentage elongation at break were potentially influenced by the filler loading and particle size. This happened due to the SiO_2_ contents in the OPBA. Similarly, Sipaut [22] reported that the tensile, flexural and thermal properties of the epoxy composite were enhanced by SiO_2_. As shown in Table 4, the epoxy polymer composite filled with a smaller particle size of OPBA (50 μm) resulted in higher tensile strength, when compared with the larger particle sizes (100 μm and 150 μm). The increase of tensile strength is possibly affected by the stress transfer between the matrix and filler particles [23]. The smaller particle size has a higher surface area that can enhance the interfacial adhesion between the filler and the matrix [24]. As a result, it provides better load transfer and thus increased the mechanical properties of the composites.

**Table 4:** Mechanical properties of OPBA filled epoxy polymer composite.

Filler loading (wt. %)	Tensile strength (MPa)			Tensile modulus (GPa)			Elongation at break (%)			Flexural modulus (GPa)			Flexural strength (MPa)			Impact strength (kJ/m2)		
	Particle size (μm)			Particle size (μm)			Particle size (μm)			Particle size (μm)			Particle size (μm)			Particle size (μm)		
	50	100	150	50	100	150	50	100	150	50	100	150	50	100	150	50	100	150
10	51.56	49.97	46.88	0.83	0.81	0.76	4.62	5.10	5.32	4.73	4.51	4.17	105.28	101.72	94.84	2.73	2.45	2.30
20	53.36	51.00	47.47	0.86	0.83	0.76	4.55	4.89	5.12	4.74	4.57	4.26	109.30	104.04	96.14	2.83	2.50	2.33
30	55.49	53.17	48.22	0.89	0.88	0.77	4.51	4.86	5.08	5.12	4.89	4.39	114.07	108.88	97.83	2.94	2.60	2.36
40	53.00	50.64	46.98	0.85	0.82	0.76	4.50	4.79	4.94	4.70	4.63	4.12	108.51	103.24	95.04	2.81	2.48	2.30
50	48.77	47.24	43.88	0.79	0.77	0.71	4.21	4.53	4.68	4.45	4.14	3.96	99.05	95.64	88.11	2.58	2.31	2.15
Neat epoxy		45.83			0.77			6.05			3.99			55			2.60	

It was observed that both tensile strength and tensile modulus were initially increased with increasing OPBA filler loading up to 30 wt.% and thereafter decreased with the loading of more than 30 wt.%. The highest tensile strength and tensile modulus were gained at 30 wt.% OPBA filler loading and 50 μm particle size at 55.49 MPa and 0.89 GPa, respectively. The increase of tensile strength and tensile modulus with increasing OPBA filler loading up to 30 wt.% was due to the increase of interfacial adhesion bonding between filler and the neat epoxy matrix [24]. However, the tensile strength and tensile modulus of the composites decreased with filler loading over 30 wt.% might due to the poor interaction between filler and polymer matrix [15], which weakening the interfacial-adhesion between the filler and the matrix. Moreover, the agglomeration of OPBA particles with an excessive amount of OPBA filler is usually the main factor that caused the premature failure of a polymer composite [25]. Thus, the tensile strength and tensile modulus were decreased. This result can is confirmed by FE-SEM (Figure 4), where rough fractured surfaces were observed at higher filler loading. Furthermore, there was more filler pulling out from the matrix at higher filler loading. This can be attributed to the decrease of the tensile strength and tensile modulus of epoxy composite filled with higher filler loading [26].

The elongation at break for the neat epoxy was determined to be 6.05%. The elongation at break was decreased to 4.62%, 5.10% and 5.32% with the incorporation of 50 μm, 100 μm and 150 μm OPBA filler in the epoxy composites, respectively. The decrease of elongation at break with the increase of particle sizes might be due to the decrease of deformability of the interface between the filler and the epoxy matrix [27]. Table 4 also shows that the elongation at the break of OPBA filled epoxy composites were further decreased with increasing filler loading (wt.%) and decreasing particle size (μm). The minimal elongation at break of OPBA filled epoxy composites was 4.21%, gained by 50 wt.% filler loading with 50 μm of particle size. The percentage of elongation at break increased with increasing particle size due to the reduction in the deformation of weak interfacial bonding between epoxy and filler matrix with larger particle size. This behaviour enhances the ductility of the composite and thus increased the elongation at break [26]. The finding was found similar to the study conducted by Husseinsyah and Mostapha [27]. The study reported that the decrease of elongation at break of the coconut shell filled polyester composites with higher filler loading attributes to the reduction of deformability between filler and composite matrix [27]. With an increasing filler loading, the crack moves to the interfacial bonding and therefore decreases the elongation at the break of the composites.

The effect of OPBA particle size and percentage filler loading on the flexural strength and flexural modulus were shown in Table 4. The influence of OPBA as a reinforcement in epoxy polymer composite on the flexural strength and flexural modulus was similar to the tensile strength and tensile modulus. Wherein, the highest flexural strength and flexural modulus were gained at 30 wt.% OPBA loading and particle size of 50 μm. This result suggested that the smaller particle size OPBA dispersed well in the polymer matrix. Therefore, the crack propagation is lengthened, and the plastic deformation of the polymer matrix is increased, which resulted in higher flexural strength of the composites [27,28].

However, the reduction of flexural strength of the composited with increasing OPBA filler loading was observed because of the declination of filler and matrix interactions due to agglomeration of OPBA fibres with excessive amount filler (Figure 4) [28]. The impact strength of microstructured OPBA reinforced epoxy polymer composites was increasing with percentage filler loading and decreased with increasing OPBA particle size from 50 μm to 150 μm (Table 4). The optimal impact strength of micro-structured OPBA reinforced epoxy composites was determined to be 2.94 kJ/m^2^ at particles size 50 μm and 30 wt.% filler loading. Generally, smaller particle size filler has a low aspect ratio compared to the larger particle size filler. However, the large particle size of filler may act as create initiation of the epoxy composite due to the stress concentration near the edges and having a large aspect ratio [29]. It is, therefore, the decrease of the impact strength of microstructured OPBA reinforced epoxy composites with increasing particle size and filler loading over 30 wt.% was observed due to the strong agglomeration of filler particles with larger particles size as can be seen in Figure 4. An excessive amount of filler loading results in the non-homogenous distribution of filler particles in the polymer matrix. Thus, the impact strength was decreased [30].

### 3.4 Thermogravimetric analysis (TGA)

**Figure 5:**
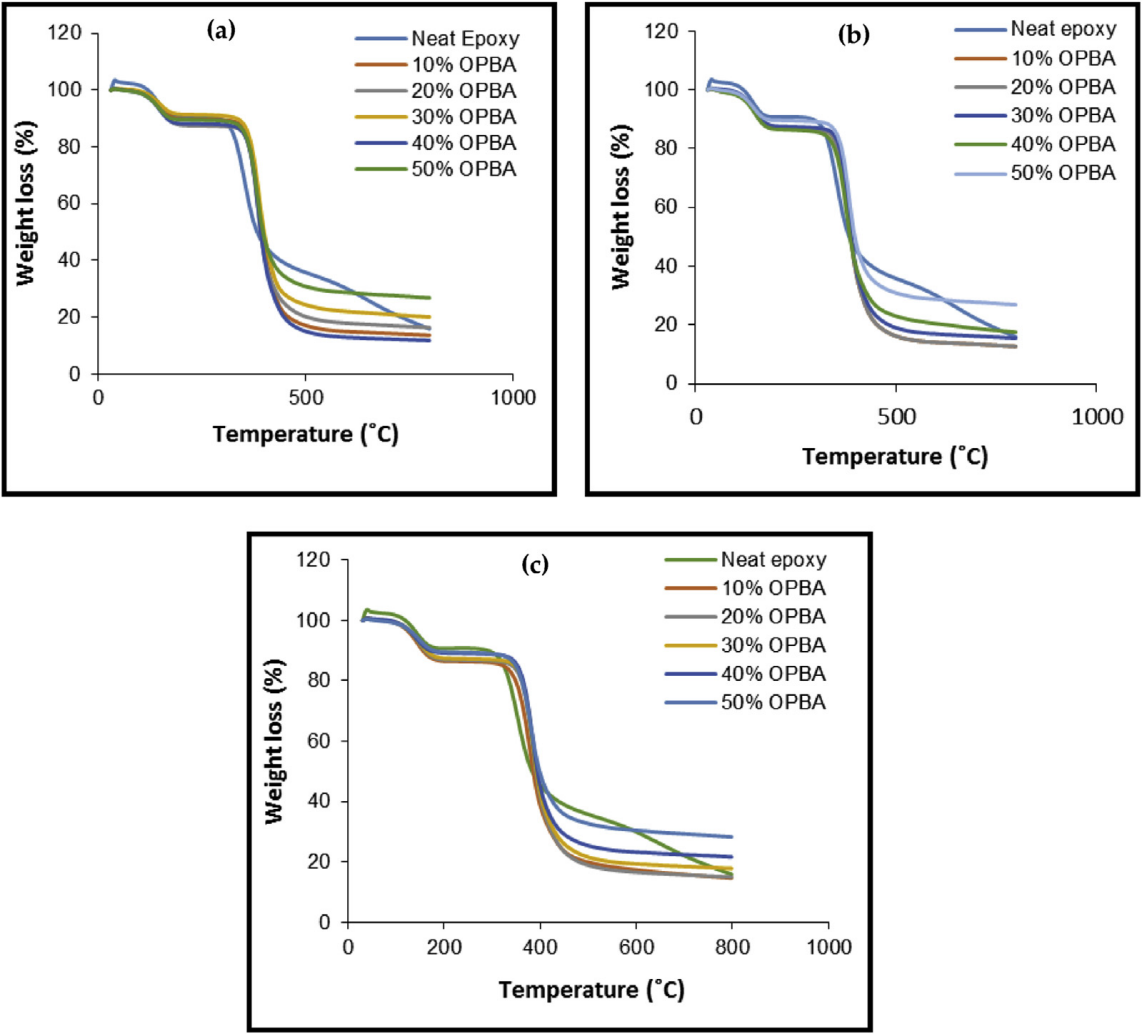
Thermogravimetric analysis of filled epoxy polymer composite in different particle sizes. (a) 50 μm, (b) 100 μm and (c) 150 μm.

Figure 5 shows the influence of the microstructured OPBA reinforcement in epoxy polymer composite on the improvement of thermal properties. The decomposition temperatures such as maximum thermal degradation temperature (T_max_), initial decomposition temperature (T_i_) and final decomposition temperature (T_f_) were determined from the TGA analysis, as summarized in Table 5. It was found that the thermal degradation temperatures such as T_i_, T_f,_ and T_max_ increased with the incorporation of OPBA filler in the composites. However, the thermal degradation temperatures slightly increased with increasing temperature and decreased with the particle size. The highest thermal degradation temperature for T_i_, T_f,_ and T_max_ was obtained with 50 wt.% OPBA filler loading and 50 μm particle size at about 364 °C, 435 °C, and 389 °C, respectively. The thermal stability increased with increasing filler loading and sizes due to the presence of higher silica content and other inorganic particles in OPBA, which potentially increased the thermal properties of the epoxy composites [31].

**Table 5:** Thermal properties of OPBA filled epoxy polymer composite.

Filler loading (wt.%)	Thermal Degradation temperature (°C)									Char Residue (%)		
	50 μm			100 μm			150 μm					
	T*i*	T_f_	T_max_	T*i*	T_f_	T_max_	T*i*	T_f_	T_max_	50 μm	100 μm	100 μm
10	360.1	420.2	385.1	348.3	420.2	382.1	349.0	415.9	377.8	11.83	12.71	14.75
20	360.6	427.7	386.0	352.3	423.3	382.3	353.3	417.8	377.9	13.71	15.52	15.08
30	360.7	432.7	386.2	353.4	424.4	387.6	354.6	421.2	381.9	16.42	17.58	17.87
40	361.3	433.3	387.5	359.0	431.2	388.2	355.7	421.9	381.9	20.13	23.61	21.72
50	363.6	434.7	389.3	360.7	433.5	389.1	361.0	427.9	383.2	23.75	26.90	28.36
Neat Epoxy	323.1	382.6	351.4							5.95		

The determination of char residue of neat epoxy and OPBA reinforced epoxy composites showed that the percentage of char residue increased with increasing OPBA filler particle size and filler loading (Table 5). This occurrence was due to the increase of non-volatile carbon content with the incorporation of OPBA filler in the composite, which further increased with the increase of OPBA particle size and filler loading [31].

### 3.5 Contact angle measurement

**Figure 6:**
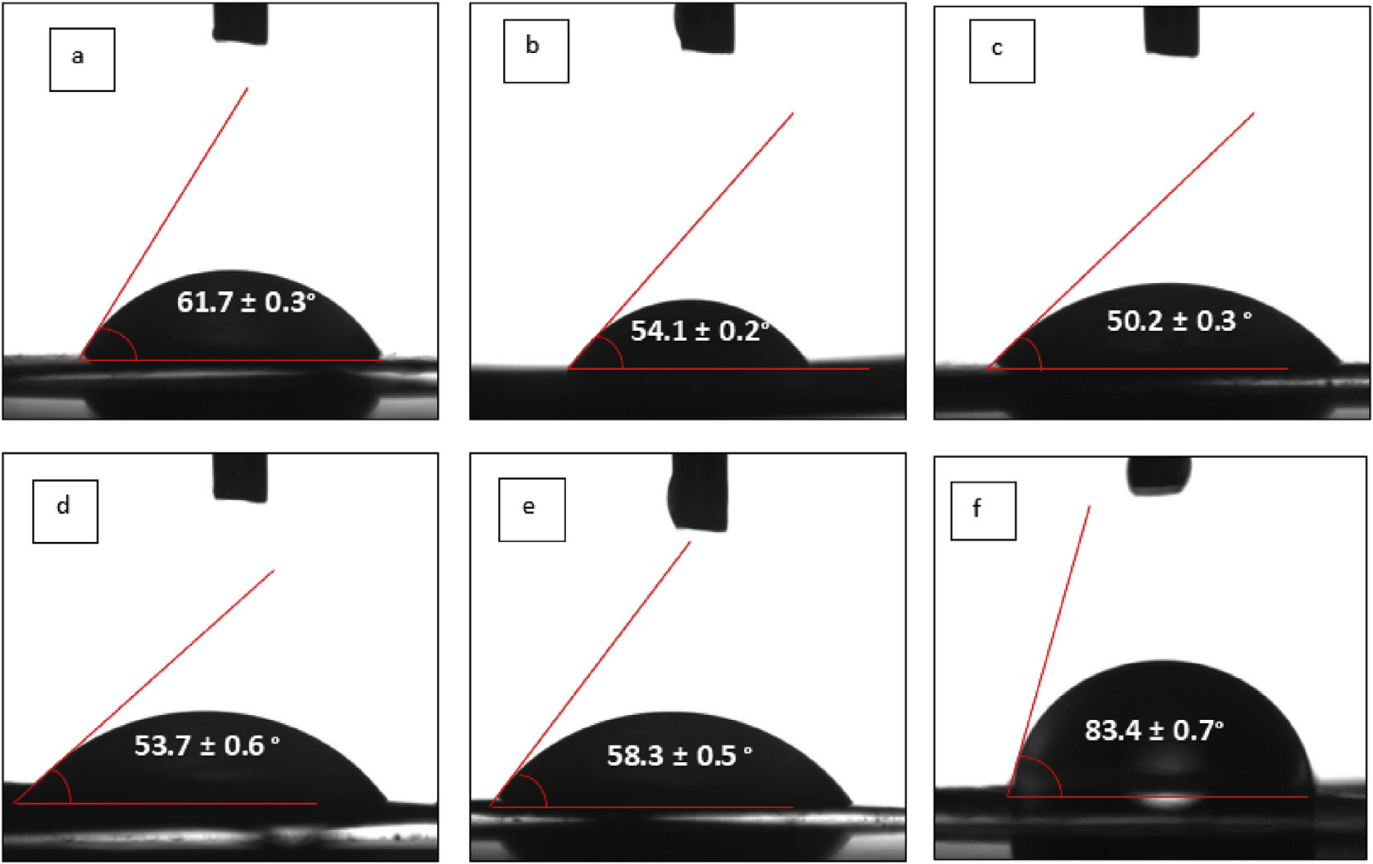
Contact angle measurements of the OPBA filled epoxy composites at OPBA particles size of 50 μm with varying percentage filler loading (a) 10 wt.%, (b) 20 wt.%, (c) 30 wt.%, (d) 40 wt.%, (e) wt.% and (f) Neat epoxy.

Table 6 shows the contact angles of the epoxy polymer composite incorporated with microstructured OPBA at varying filler loadings and particle sizes. The presence of microstructured OPBA has led to a noticeable decrease in the contact angle values of the composites. The contact angle of the neat epoxy was found at 83.4° and it was reduced with the increase of OPBA particle sizes from 50 μm to 150 μm. Similarly, the contact angle of OPBA reinforced epoxy composites further reduced with increasing incorporation of filler loading from 10 wt.% to 30 wt.% and thereafter increased, as shown in Figure 6. The minimal contact angle was gained by the OPBA particle size of 50 μm and 30 wt.% filler loading at 50.2°. The decrease of the contact angle of the OPBA reinforced epoxy composites up to 30 wt.% filler loading is attributed to the optimal chemical compatibility between epoxy and OPBA particles [32]. The deteriorating of the dispersion degree of the micro filler at higher filler concentrations, possibly forming microparticle agglomerates, which caused the contact angles to increase at higher filler loading (ie: from 30 wt.% filler loading onwards) [33].

**Table 6:** Contact angle measurements of the OPBA filled epoxy composites.

Filler loading (wt.%)	Contact Angle (°)		
	50 μm	100 μm	150 μm
10	61.7	63.2	76.2
20	54.1	59.9	71.7
30	50.2	55.3	60.5
40	53.7	59.4	63.1
50	58.3	62.1	74.4
Neat epoxy	83.4

Based on the finding of the present study it can be concluded that both percentage OPBA loading and its particle size play an important role in influencing the physical, mechanical and thermal properties of the composite. It was found that tensile strength and tensile modulus were increased with increasing OPBA filler loading up to 30 wt.% and decreased with further increasing the percentage OPBA loading, whereas tensile strength and tensile modulus decreased with increasing particle size. The highest tensile strength and tensile modulus were gained at 30 wt.% OPBA filler loading and smallest particle size (50 μm) at 55.49 MPa and 0.89 GPa, respectively. A similar pattern was noticed in flexural modulus, flexural strength, and impact strength. However, the elongation at break of OPBA filled epoxy composites decreased with increasing filler loading (wt.%) and decreasing particle size (μm) might due to the reduction in the deformation of weak interfacial bonding between epoxy and filler matrix. On the other hand, the thermal stability increased with increasing filler loading and OPBA particle size due to the presence of higher silica content and other inorganic particles. The contact angle was achieved by OPBA particle size of 50 μm and 30 wt.% filler loading showed the minimal contact angle at 50.2° due to the optimal chemical compatibility between epoxy and OPBA particles. In short, the results revealed that the incorporation of OPBA in the epoxy matrix has successfully enhanced the physical, mechanical and thermal properties of the epoxy polymer composite.

## 4 Conclusions

In this study, the influence of micro-structure OPBA as a source of inorganic silica on the physical, mechanical and thermal properties of OPBA reinforced epoxy composite was determined. The chemical composition analyses OPBA showed that the OPBA contained a high amount of silica, which is an account of about 54 wt.%. The incorporation of the OPBA enhances the physical, mechanical and thermal properties of the epoxy matrix The OPBA filled composite at 30 wt.% filler loading and 50 μm particle exhibited the highest physical and mechanical properties mainly due to good interfacial adhesion formed between the filler and the matrix. The findings of the present study revealed that the OPBA filler has potentially enhanced the physical, mechanical and thermal properties of epoxy polymer composites.

## Declarations

### Author contribution statement

Samsul Rizal: Conceived and designed the experiments; Contributed reagents, materials, analysis tools or data.

H. M. Fizree, Deepu A. Gopakumar, Eunice Chong Wan Ni: Performed the experiments; Analyzed and interpreted the data.

Md. Sohrab Hossain: Conceived and designed the experiments; Wrote the paper.

Ikramullah: Analyzed and interpreted the data; Contributed reagents, materials, analysis tools or data.

H. P. S. Abdul Khalil: Conceived and designed the experiments; Contributed reagents, materials, analysis tools or data; Wrote the paper.

### Funding statement

This work was supported by Ministry Research, Technology and Higher Education of the Republic of Indonesia by World Class Professor (WCP) (No T/46/D2.3/KK.04.05/2019).

### Competing interest statement

The authors declare no conflict of interest.

### Additional information

No additional information is available for this paper.
